# Topological resilience of EEG-based functional networks during virtual reality-induced emotional states

**DOI:** 10.3389/fnhum.2026.1777965

**Published:** 2026-04-10

**Authors:** Min Jae Lee, Gang Wang, Won Hee Lee

**Affiliations:** 1 Department of Artificial Intelligence, Kyung Hee University, Yongin-si, Republic of Korea; 2 Key Laboratory of Biomedical Information Engineering, Ministry of Education, Institute of Biomedical Engineering, School of Life Science and Technology, Xi'an Jiaotong University, Xi'an, China; 3 Department of Software Convergence, Kyung Hee University, Yongin-si, Republic of Korea

**Keywords:** EEG, emotion processing, functional brain networks, network resilience, virtual reality

## Abstract

**Introduction:**

Virtual reality (VR) provides an immersive environment for inducing emotional experiences, offering a naturalistic framework for investigating brain network dynamics. However, traditional emotional neuroscience has largely focused on regional activations, leaving the topological robustness and adaptive capacity of integrated brain networks underexplored. This study addresses this gap by applying a graph-theoretical framework to quantify how different emotional states modulate the resilience of functional architectures against systematic disruptions.

**Methods:**

In this study, we examined the resilience of EEG-based functional brain networks during negative, neutral, and positive emotional states induced by VR stimuli. Functional connectivity was computed using coherence across six frequency bands (delta to high gamma), and graph-theoretical measures were applied to characterize network topology. To assess resilience, we simulated network disruptions using two complementary approaches: targeted attacks (removing high-centrality nodes) and random failures (removing nodes randomly). Changes in global efficiency and the largest connected component were tracked as nodes were progressively removed.

**Results:**

Our findings revealed emotion-specific resilience profiles. In the alpha band, both negative and positive emotions demonstrated enhanced resilience to targeted attacks compared to neutral states, maintaining higher efficiency and greater network integrity, with positive emotions showing particularly strong preservation of large-scale connectivity. In the high gamma band, networks during negative emotional states exhibited greater robustness than those during positive emotions, indicating enhanced capacity to withstand targeted disruptions.

**Discussion:**

These findings suggest that emotional experiences are associated with differences in functional brain architecture that affect network robustness and adaptability, providing insights into neural mechanisms of emotion regulation and potential applications for emotion-aware VR systems.

## 1 Introduction

Understanding how the human brain adapts and maintains functional integrity under emotionally evocative conditions is fundamental to advancing affective neuroscience and mental health research (Wang et al., 2020). Emotional experiences trigger widespread, dynamic changes in neural activity that profoundly influence core cognitive processes including perception (Phelps et al., 2006), decision-making (Mitchell, 2011), and memory formation (Richter-Levin and Akirav, 2003). These emotional modulations extend beyond isolated brain regions, engaging distributed networks that must coordinate rapidly to support adaptive behavioral responses. Virtual reality (VR) has emerged as a transformative tool for emotion research, providing immersive, multisensory environments that closely approximate real-world emotional experiences while maintaining the experimental control necessary for rigorous scientific investigation (Marín-Morales et al., 2020). When integrated with electroencephalography (EEG), VR enables continuous, high-temporal-resolution monitoring of brain dynamics during naturalistic emotional states, offering unprecedented opportunities to investigate the neural mechanisms underlying emotional processing as they unfold in ecologically valid contexts (Hofmann et al., 2021; Yu et al., 2022). This VR-EEG integration addresses a critical limitation of traditional emotion research paradigms that rely on passive stimulus observation, allowing researchers to capture the complexity of emotional responses as they occur during active engagement with immersive environments.

Despite significant advances in emotion research, the resilience of brain networks, defined as a neural system's capacity to maintain functional organization and recover from perturbations (Paban et al., 2019), remains underexplored in emotional contexts, particularly within immersive VR environments. Traditional approaches have predominantly focused on identifying regional activation patterns or quantifying mean connectivity differences across emotional states (Kassam et al., 2013; Lee and Hsieh, 2014; Baumgartner et al., 2008; Rodríguez et al., 2015), often treating the brain as a collection of isolated regions rather than examining the topological robustness and adaptability of integrated network architectures. This approach overlooks a fundamental property of neural systems: their ability to maintain global functional coherence when faced with disruptions or increased processing demands. Understanding how brain networks preserve structural integrity and information flow during emotionally challenging or salient events is essential for elucidating the neural mechanisms underlying emotional processing, regulation, and resilience. Moreover, network resilience may represent a key marker of emotional health, with implications for understanding vulnerability to affective disorders and predicting individual differences in emotional regulation capacity. The immersive nature of VR environments provides an ideal testbed for investigating network resilience under naturalistic emotional conditions, as it allows examination of how networks reorganize and adapt during sustained, ecologically valid emotional experiences rather than brief, isolated stimulus presentations.

To address this gap, we adopt a graph-theoretical framework to systematically assess the resilience of EEG-derived functional brain networks during emotional experiences elicited in immersive VR environments (Albert et al., 2000; Rubinov and Sporns, 2010; Lynall et al., 2010). Graph theory offers a powerful mathematical toolkit for quantifying fundamental network properties including integration (global information transfer efficiency), segregation (specialized local processing), and centrality (identification of critical hub regions) (Sporns, 2018). These metrics enable characterization of both the structural organization and functional efficiency of complex brain networks. Recent advances in network resilience analysis, including simulated targeted node removal (attacking critical hubs), random node failure (modeling stochastic disruptions), and damage propagation assessment (Joyce et al., 2013), enable systematic evaluation of how efficiently networks maintain connectivity and reorganize following progressive degradation (Joyce et al., 2013; Gonzalez-Escamilla et al., 2018). Critically, different emotional states may alter network topology in ways that enhance or diminish resilience to disruption, reflecting distinct strategies for maintaining functional coherence under varying affective demands. Applying these network resilience methodologies to emotion research offers valuable mechanistic insights into how emotional states modulate the brain's capacity for robust information processing and adaptive network reorganization (Cisler et al., 2013; Fischer et al., 2018), potentially revealing fundamental principles of emotional regulation and vulnerability to affective disturbances.

In this study, we investigate how different emotional states (negative, neutral, and positive) influence brain network resilience using coherence-based functional connectivity derived from high-density EEG data during immersive VR experiences. Leveraging VR's ecological validity to elicit naturalistic emotional responses, we decompose EEG signals into six frequency bands (delta to high gamma) and construct functional brain networks for each emotional condition. To systematically assess network resilience, we employ both targeted node removal strategies (based on betweenness centrality to simulate attacks on critical hub regions) and random node failure protocols (to simulate stochastic disruptions). We quantify resilience through changes in key graph-theoretical metrics—global efficiency, size of the largest connected component, and degree of network fragmentation—as networks undergo progressive degradation. By comparing these resilience profiles across emotional states and frequency bands, we aim to identify emotion-specific vulnerabilities and adaptive features in brain network topology that reflect the brain's dynamic capacity to maintain functional integration under emotional demands. This work contributes to the expanding field of brain network resilience research and its applications in affective neuroscience. We present a novel methodological approach for quantifying functional brain network robustness during emotionally charged experiences, while highlighting the synergistic potential of EEG and VR technologies for real-time emotional processing investigations. This study makes several key contributions: (1) providing the first systematic investigation of brain network resilience across emotional states in immersive VR environments; (2) revealing frequency-specific emotion-resilience dissociations where positive emotions enhance alpha-band network integration while negative emotions fortify high-gamma localized processing; (3) demonstrating that emotional modulation of resilience is strategically organized around critical hub nodes rather than uniformly distributed; and (4) establishing methodological feasibility for network resilience analysis in ecologically valid emotional contexts with translational implications for understanding affective disorders.

## 2 Materials and methods

### 2.1 Dataset

This study utilized the VREED dataset, which contains multi-channel EEG recordings from 25 participants (mean age = 22.92 years; SD = 1.38; 10 females) exposed to immersive 3D virtual reality stimuli (Yu et al., 2022). All participants had normal or corrected-to-normal vision and no history of psychiatric or major medical conditions, as confirmed by screening according to institutional protocols. The experiment was approved by the local ethics committee, and written informed consent was obtained from all participants.

Participants viewed sixty 4-s 3D VR videos (4096 × 2048 resolution, 30 fps) depicting Shanghai landmarks (e.g., the Bund, Oriental Pearl), street scenes, and school-themed events through an HTC Vive headset. The videos were categorized into three emotional classes—positive, neutral, and negative (20 videos each). Each subject completed 120 trials comprising two 30-video subsets: one containing 20 positive + 10 neutral clips, and another with 20 negative + 10 neutral clips, each repeated twice. After each trial, participants rated emotional valence on a 1–9 scale (Russell, 1980; Bradley and Lang, 2007).

EEG signals were recorded using a 64-channel cap following the international 10–20 system (Jurcak et al., 2007). After removing EOG and EMG channels, 59 channels covering frontal, central, occipital, and bilateral temporal regions were retained for analysis. Recordings were acquired at 1,000 Hz for 4 s per trial. Data preprocessing was carried out in EEGLab (Delorme and Makeig, 2004) using the following pipeline: (1) removing artifacts and power frequency interference to ensure signal integrity; (2) baseline adjustment to account for potential drifts in EEG potentials; (3) cutting bad trials containing excessive noise or signal instability; and (4) excluding participants with poor data quality (6 out of 25 participants) to maintain high data robustness, resulting in a final dataset of 19 valid participants. Data from six participants were excluded due to poor signal quality, leaving 19 subjects (mean age = 22.84 years; SD = 1.50; 6 females) for final analysis. In total, 2,043 trials were analyzed (positive = 708, neutral = 639, negative = 696). Full details of the original VREED acquisition protocol and preprocessing pipeline are described in (Yu et al., 2022).

### 2.2 Network construction

**Figure 1:**
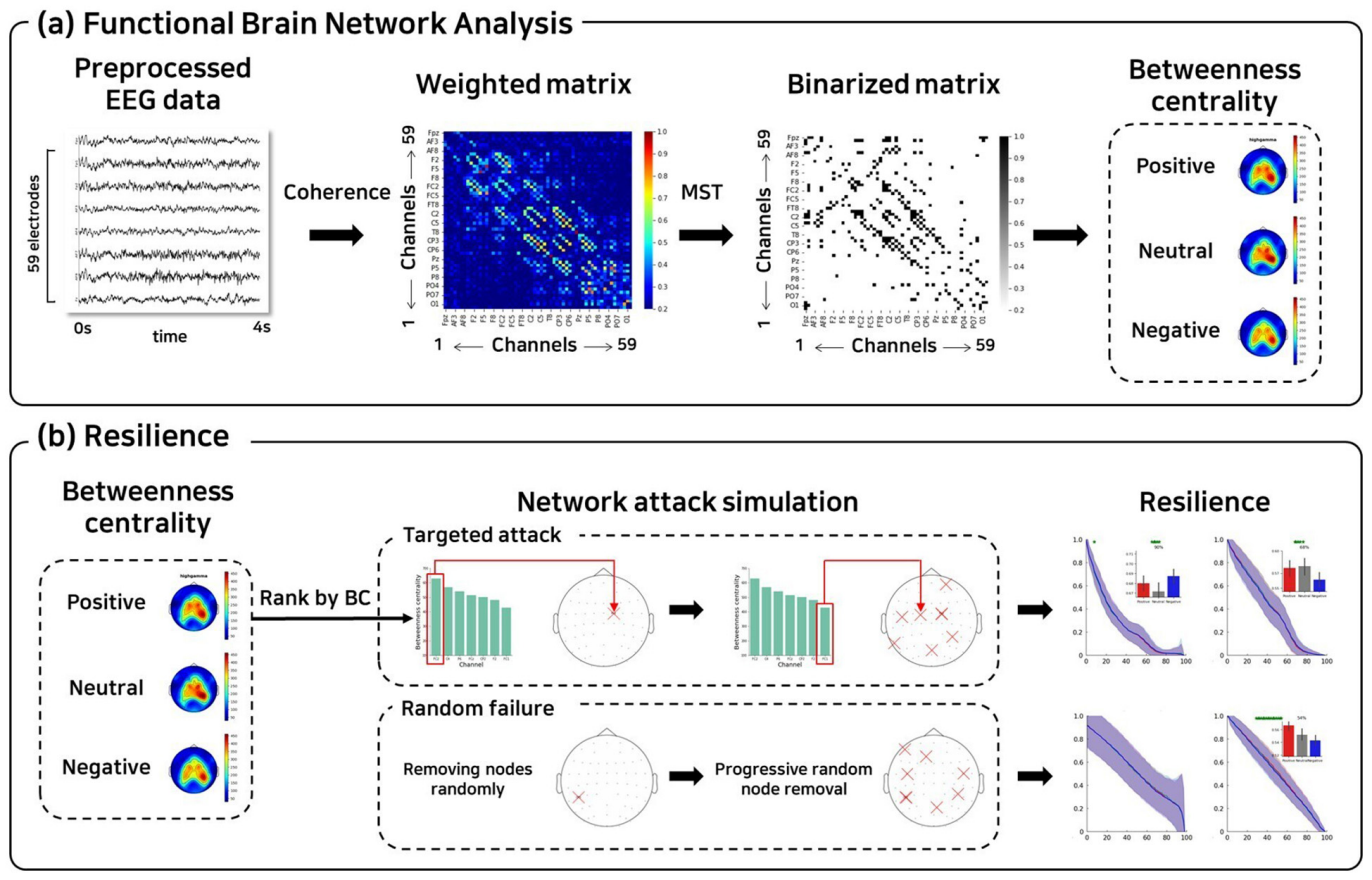
Overview of the analysis pipeline. **(a)** Spectral coherence was computed from 59-channel EEG signals to construct undirected weighted connectivity matrices. These matrices were converted into undirected binary graphs using a minimum spanning tree (MST) algorithm, from which betweenness centrality was derived. **(b)** Network resilience was evaluated under targeted attacks and random failures through sequential node removal. In targeted attacks, nodes were removed in descending order of betweenness centrality, whereas in random failures, nodes were removed randomly. After each removal, global network efficiency was recalculated to quantify changes in network robustness.

In this study, the EEG-based functional connectivity (FC) network was modeled as a graph consisting of nodes (EEG electrodes) and edges (the pairwise associations between EEG signals from different channels) (Ismail and Karwowski, 2020) (Figure 1a). To estimate the FC between channel pairs, coherence was employed since it is a widely accepted measure of phase synchrony between two EEG signals (Ismail and Karwowski, 2020; Liu et al., 2019; Bowyer, 2016). We selected coherence because it provides interpretable frequency-specific coupling strength and facilitates comparison with existing emotion recognition literature (Basharpoor et al., 2021; Aljalal et al., 2022; Bevilacqua et al., 2019). However, we acknowledge that coherence is susceptible to spurious connectivity from volume conduction and common reference effects, where activity from a single neural source appears at multiple scalp electrodes, creating inflated zero-lag correlations (Bastos and Schoffelen, 2015). Alternative connectivity measures such as weighted phase lag index (wPLI) or imaginary coherence reduce sensitivity to these artifacts by excluding instantaneous correlations (Vinck et al., 2011; Nolte et al., 2004). Nevertheless, our focus on relative differences in network topology across emotional conditions, rather than absolute connectivity localization, may partially mitigate these concerns, as volume conduction artifacts should affect all conditions similarly under stable recording conditions. Specifically, coherence quantifies the degree of consistency in both amplitude and phase relationships between two signals [x(t) and y(t)] in the frequency domain, and is defined as:


$$C_{XY}\left(\lambda \right)=\frac{{\left|P_{XY}\left(\lambda \right)\right|}^{2}}{P_{XX}\left(\lambda \right)P_{YY}\left(\lambda \right)}$$

where *C*_*XY*_(λ) denotes the coherence between x(t) and y(t) at frequency λ. *P*_*XX*_(λ) and *P*_*YY*_(λ) represent the power spectral densities of x(t) and y(t), respectively, while *P*_*XY*_(λ) indicates their cross-power spectral density, reflecting the shared spectral power between the two signals.

Coherence values range from 0 to 1, with higher values signifying stronger phase synchrony between signal pairs. Connectivity was computed across six standard EEG frequency bands: delta (0.5–4 Hz), theta (4–8 Hz), alpha (8–13 Hz), beta (13–30 Hz), gamma (30–50 Hz), and high-gamma (50–80 Hz). For each trial and frequency band, an FC matrix of size (59 × 59) was generated, representing all pairwise functional associations among the 59 EEG electrodes.

### 2.3 Minimum spanning tree

To reduce network complexity and extract the most informative connections from the weighted undirected FC networks, we applied the minimum spanning tree (MST) algorithm (Achard and Bullmore, 2007) (Figure 1a). MST provides a principled, threshold-free approach to network construction that ensures connectedness and has been widely used in neuroscience (He et al., 2008; Fornito et al., 2016). This approach is particularly valuable for resilience studies because it: (1) eliminates arbitrary threshold selection that could confound cross-condition comparisons, (2) identifies the network's critical backbone connectivity with minimal redundancy, and (3) produces standardized network topologies (exactly N-1 edges, where N is the number of nodes) that enable fair comparison of resilience across emotional states without confounding effects from differences in network density or thresholding. For each FC matrix, connection strengths (link weights) were first ranked in descending order. Using Kruskal's algorithm (Lee et al., 2017), the edges with the highest weights were iteratively added until all nodes were connected, yielding a tree structure with the minimal number of edges (E = N – 1), where N and E denote the number of nodes and edges, respectively, and ensuring the absence of cycles. The resulting MST was then represented as a binary adjacency matrix, where existing connections were assigned a value of 1 and absent connections were assigned 0. This procedure produced binary FC matrices with an average connection density of approximately 8%, retaining only the most salient functional connections for subsequent graph-theoretical analysis. However, we acknowledge that MST enforces an acyclic tree topology that eliminates redundant pathways and cycles—features critical to functional brain network resilience (Cabral et al., 2012). This structural constraint may influence resilience measurements, as the removal of any node in a tree necessarily fragments the network. To assess sensitivity to this methodological choice, we conducted supplementary analyses using proportional thresholding (retaining top 8% of edges without MST constraint), which yielded qualitatively consistent emotional state differences (see Supplementary materials).

### 2.4 Network resilience

To evaluate the resilience of brain networks against simulated disruptions, we performed network attack simulations on the FC matrices (Figure 1b). Two node-removal strategies were considered: (1) a targeted attack, in which nodes with the highest betweenness centrality were sequentially removed, and (2) a random failure, in which nodes were eliminated in a random order (Albert et al., 2000), as illustrated in Figures 1a, b. Nodes were progressively removed in 1% increments, ranging from 0% (intact network) to 100% (complete removal). After each removal step, two network metrics were computed, namely the size of the largest connected component and global efficiency, to assess how the network structure deteriorated under increasing disruption. We selected these complementary metrics because global efficiency quantifies functional integration (how efficiently information flows across the network), while largest connected component (LCC) measures structural integrity (whether the network remains connected or fragments) (Albert et al., 2000; Achard and Bullmore, 2007; He et al., 2008). Together, these metrics provide foundational characterization of network robustness and have been widely validated in resilience research (Fornito et al., 2016; Lee et al., 2017). While additional metrics such as modularity changes, clustering coefficient degradation, or composite robustness indices could provide further insights, we prioritized these two well-established measures with clear functional relevance to emotional processing (Rubinov and Sporns, 2010). Network robustness was assessed by analyzing the rate of decline in these metrics under increasing levels of disruption. Networks that maintained a relatively large connected component and high global efficiency despite substantial node loss were considered to exhibit greater resilience (Lynall et al., 2010; Cabral et al., 2012; Bassett et al., 2008; Lo et al., 2015).

### 2.5 Statistical analysis

We compared network resilience measures across the three emotional conditions (negative, neutral, positive) using one-way analysis of variance (ANOVA) for each frequency band. When ANOVA revealed significant differences, post-hoc pairwise comparisons were performed using Games-Howell tests to identify which condition pairs differed significantly (Kim, 2017; Games and Howell, 1976). Multiple comparisons were corrected using the false discovery rate (FDR) method (Benjamini and Hochberg, 1995), with a corrected *p* < 0.05 considered statistically significant. In addition, we computed effect sizes (paired Cohen's d based on area-under-curve summaries of the resilience trajectories) with 95% confidence intervals to quantify the magnitude and uncertainty of observed differences between emotional conditions, providing transparency regarding the strength of evidence given the modest sample size (*N* = 19) (Cohen, 1977).

## 3 Results

### 3.1 Frequency-specific patterns

Analysis across all six frequency bands (delta, theta, alpha, beta, low gamma, high gamma) revealed that emotional modulation of network resilience was most pronounced in the alpha and high gamma bands (Supplementary Figures S2, S3), with minimal effects observed in delta and theta bands under both perturbation conditions. Beta and low gamma bands showed intermediate patterns, with modest but significant emotional effects under targeted attack but not under random failure (p > 0.05). These frequency-specific patterns suggest that emotional states selectively reorganize networks operating at specific temporal scales relevant to attention control and sensory processing.

### 3.2 Alpha band resilience under targeted attack

**Figure 2:**
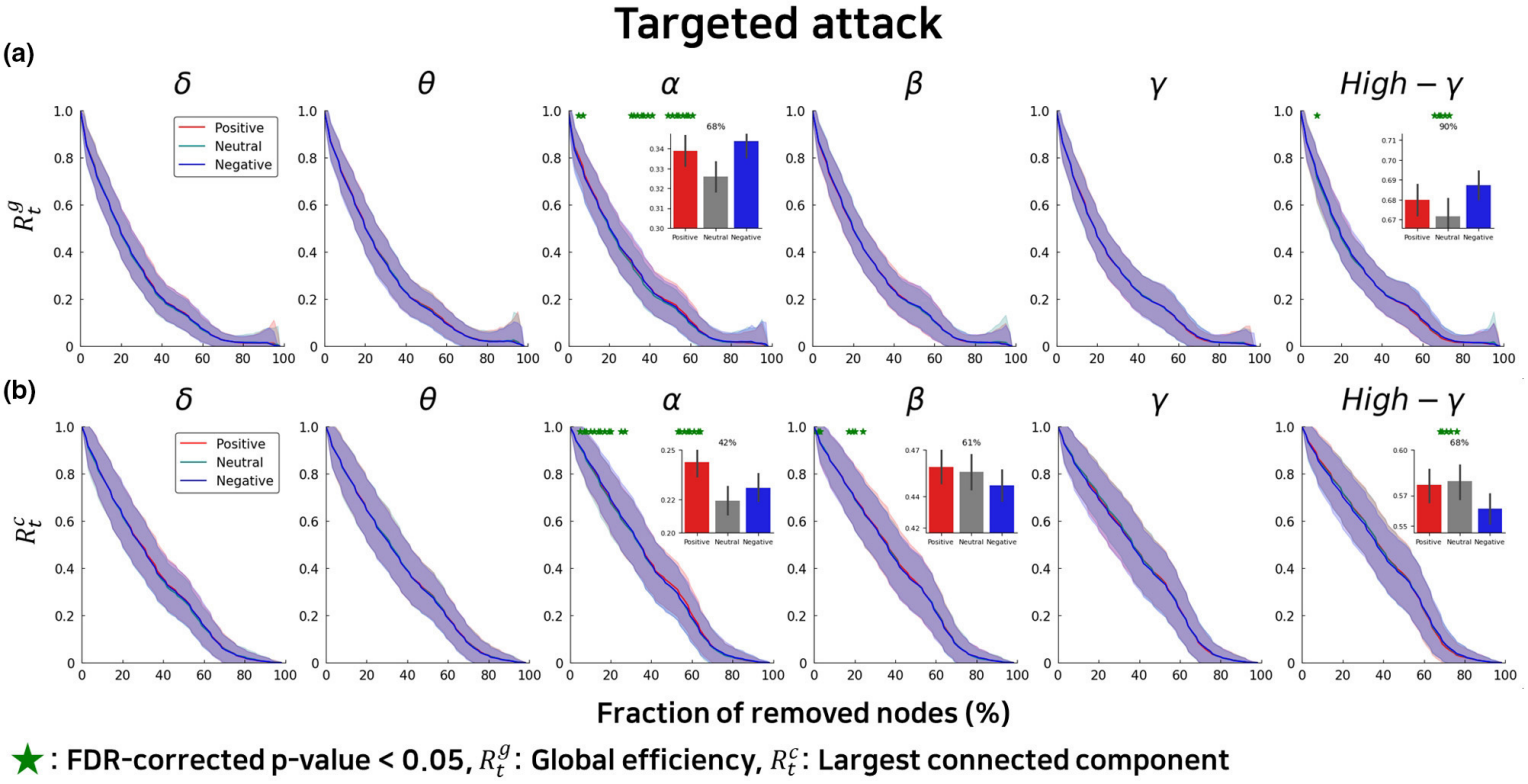
Network resilience under targeted attacks varies across emotional states and frequency bands. Resilience profiles comparing positive (red), negative (blue), and neutral (black) emotional states during progressive targeted removal of high-betweenness centrality nodes across six frequency bands (δ: delta, θ: theta, α: alpha, β: beta, γ: gamma, High-γ: high gamma). **(a)** Global efficiency ($R_{t}^{g}$) quantifies network integration and information transfer efficiency as a function of nodes removed. **(b)** Largest connected component size ($R_{t}^{c}$) indicates network fragmentation resistance. Green dots mark fractions of removed nodes where emotional states show statistically significant differences (FDR-corrected *p* < 0.05). Inset panels display detailed comparisons at specific removal fractions (%) where the most pronounced significant differences between emotional conditions are observed. Error bands represent the standard deviation across participants.

In the alpha band, emotional states modulated network resilience under targeted attack conditions (see Supplementary Figures S2, S3 for details). Negative emotions were associated with enhanced network resilience compared to neutral states, as evidenced by higher global efficiency [Δ = 31–39%, *p* < 0.05, FDR-corrected; d = 0.28, 95% CI (−0.21, 0.77)] and larger connected components [Δ = 31–44%, *p* < 0.05, FDR-corrected; d = 0.23, 95% CI (−0.26, 0.72)] across the range of node removal percentages (Figure 2). This enhancement was most pronounced at intermediate levels of disruption (31–39% and 49–61% node removal), where networks under negative emotional states maintained functional connectivity pathways that had already fragmented in neutral conditions. Positive emotions also demonstrated stronger alpha-band resilience relative to neutral states, with greater preservation of network integrity than negative emotions. Specifically, positive emotional states exhibited higher global efficiency [Δ = 37–61%, *p* < 0.05, FDR-corrected; d = 0.49, 95% CI (−0.02, 1.00)] and larger connected components [Δ = 37–43 and 49–61%, *p* < 0.05, FDR-corrected; d = 0.32, 95% CI (−0.17, 0.82)] compared to neutral conditions under targeted attack (Figure 2). The overall resilience curves for positive emotions showed a more gradual decline, indicating sustained resistance to network fragmentation even as critical hub nodes were systematically removed.

### 3.3 High gamma band resilience under targeted attack

In contrast to the alpha band, the high gamma band exhibited a distinct resilience profile across emotional states (see Supplementary Figures S2, S3 for details). Networks during negative emotional experiences demonstrated greater resilience than those during positive emotions under targeted attack, as reflected in higher global efficiency [Δ = 66–73%, *p* < 0.05, FDR-corrected; d = 0.26, 95% CI (−0.23, 0.75)] and larger connected components [Δ = 68–75%, *p* < 0.05, FDR-corrected; d = 0.24, 95% CI (−0.25, 0.73)] throughout the disruption process (Figure 2). This pattern suggests that negative emotions reorganize high gamma networks to prioritize robustness of localized, high-frequency processing streams. The divergence between negative and positive emotional states became increasingly pronounced as more nodes were removed, with negative emotions preserving critical pathways that collapsed under positive emotional conditions. Neutral states in the high gamma band showed intermediate resilience levels, differing in some ranges from both negative (*p* < 0.05) and positive (*p* < 0.05) conditions at higher disruption levels (Δ = 66–73%).

### 3.4 Resilience under random failure

**Figure 3:**
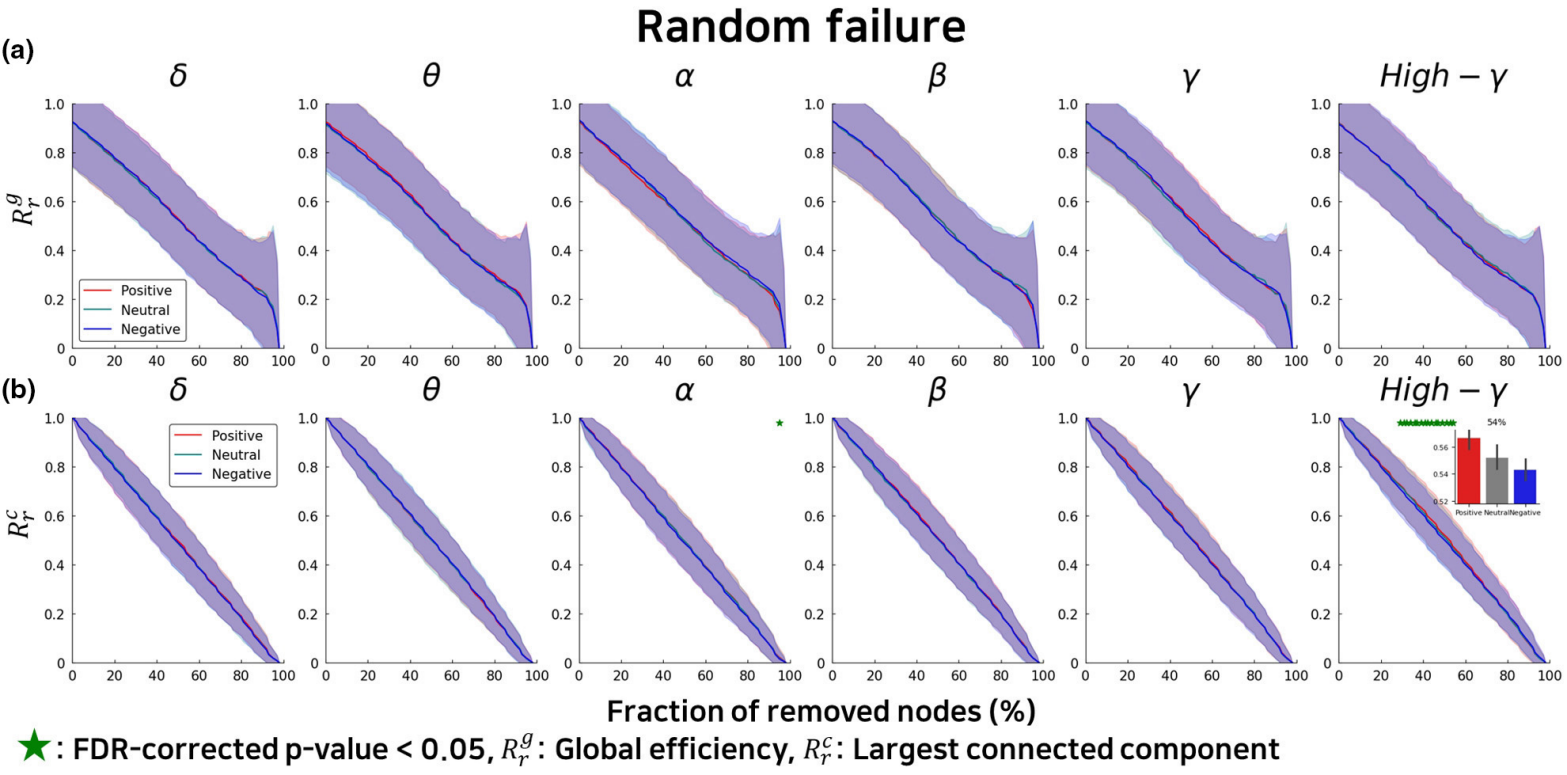
Network resilience under random failure shows distinct patterns across emotional states and frequency bands. Resilience profiles comparing positive (red), negative (blue), and neutral (black) emotional states during progressive random node removal across six frequency bands (δ: delta, θ: theta, α: alpha, β: beta, γ: gamma, High-γ: high gamma). **(a)** Global efficiency ($R_{r}^{g}$) quantifies network integration and information transfer efficiency as nodes are randomly removed. **(b)** Largest connected component size ($R_{r}^{c}$) indicates resistance to network fragmentation. Green dots mark fractions of removed nodes where emotional states show statistically significant differences (FDR-corrected *p* < 0.05). Inset panels display detailed comparisons at specific removal fractions (%) where the most pronounced significant differences between emotional conditions are observed. Error bands represent the standard deviation across participants.

Under random failure conditions, the differences in network resilience across emotional states were substantially attenuated compared to targeted attack scenarios. While the overall pattern of declining global efficiency and connected component size was similar across all emotional conditions, the magnitude of emotional modulation was markedly reduced (Figure 3). Although the alpha band was slightly higher in the emotional state than in the neutral state, the difference was not statistically significant. Furthermore, the overall magnitude of these differences was considerably smaller than that observed under targeted attack conditions. Similarly, in the high gamma band, the divergence between negative and positive emotional states was diminished under random failure, with no statistically significant contrasts detected. This asymmetry, with pronounced condition-specific effects under targeted attacks but minimal or absent effects under random failures, indicates that emotional states specifically influence the hierarchical organization and strategic importance of hub nodes rather than uniformly altering overall network connectivity.

## 4 Discussion

### 4.1 Main findings

The present study demonstrates that emotional states are associated with systematic differences in brain network resilience to structural perturbations, with distinct effects across frequency bands and disruption types. Moving beyond traditional analyses of individual regional activations, our network-level investigation revealed that alpha band networks during both positive and negative emotional states exhibited enhanced resilience compared to neutral conditions, particularly under targeted attacks. These findings suggest that emotional engagement, regardless of valence, is accompanied by greater capacity to preserve functional integration when critical network hubs are compromised (Lo et al., 2015; Cohen and D'Esposito, 2016). The observed resilience differences in the alpha band aligns with converging evidence that alpha oscillations (8–13 Hz) support large-scale network coordination and cognitive control, particularly during emotionally salient processing (Sadaghiani and Kleinschmidt, 2016; Palva and Palva, 2011). The alpha rhythm's established role in orchestrating long-range cortical communication may be particularly engaged during emotional experiences to maintain network coherence despite disruption.

Importantly, networks during positive emotions demonstrated superior alpha band resilience, surpassing both negative and neutral states. This heightened robustness likely reflects the broader cognitive and attentional resources recruited during positive affective experiences, consistent with theoretical frameworks suggesting that positive emotions are associated with expanded neural integration and processing flexibility (Isen et al., 1991). The enhanced network redundancy observed during positive emotional states could provide multiple alternative pathways for information flow, ensuring maintained global communication even when critical hub regions are removed. This adaptive network architecture may support the cognitive broadening effects associated with positive affect, potentially enabling more flexible information processing and creative problem-solving, though direct behavioral validation of this relationship was not examined in the present study. In contrast, networks during negative emotional states exhibited significantly greater resilience than positive states in the high gamma band, revealing a frequency-specific dissociation in emotion-resilience relationships. However, high-gamma findings should be interpreted cautiously given EEG limitations at high frequencies, including susceptibility to muscle artifact contamination and reduced signal reliability (Muthukumaraswamy, 2013). Given that high gamma activity is closely associated with localized processing and cortical excitation (Crone et al., 2006; Ray and Maunsell, 2011a; Buzsáki and Wang, 2012), this pattern may reflect heightened vigilance or compensatory reorganization during aversive emotional processing. The increased robustness of high-frequency networks observed under negative emotions could represent an adaptive mechanism that prioritizes maintenance of critical sensory and attentional pathways in potentially threatening environments, ensuring sustained information processing even under suboptimal conditions (Cao et al., 2020; Balconi and Lucchiari, 2008; Martini et al., 2012), though this adaptive interpretation requires validation through behavioral correlates.

The divergent resilience patterns between alpha and high gamma bands reveal that networks during different emotional states exhibit frequency-specific differences in network architecture. Networks during positive emotions display stronger alpha band resilience, potentially facilitating broad integration and flexible communication, while networks during negative emotions show selectively enhanced high gamma network stability that may preserve localized processing capabilities (Lo et al., 2015; Cohen and D'Esposito, 2016; Crone et al., 2006; Ray and Maunsell, 2011a; Buzsáki and Wang, 2012). These opposing patterns likely reflect fundamentally different computational strategies. Alpha oscillations support top-down control and long-range cortical communication (Klimesch et al., 2007; Palva and Palva, 2007), and enhanced alpha resilience during positive emotions may facilitate cognitive broadening (Fredrickson, 2001), potentially enabling flexible exploration and integration of diverse information through multiple alternative communication pathways, though this functional relationship requires validation through concurrent behavioral assessments. Conversely, high-gamma activity reflects localized cortical processing and sensory binding (Fries, 2009; Ray and Maunsell, 2011b), and enhanced high-gamma resilience during negative emotions may represent adaptive attentional narrowing that prioritizes detailed threat detection (Fredrickson and Branigan, 2005), ensuring critical sensory pathways remain functional under disruption. This frequency-dependent specialization may represent complementary adaptive strategies whereby networks reconfigure to match the specific computational and behavioral demands characteristic of different emotional contexts. The alpha-gamma dissociation suggests that network topology during emotional processing does not simply strengthen or weaken networks uniformly, but rather differs in frequency-specific ways that align with emotion-relevant cognitive and perceptual demands, though direct validation of these functional consequences through behavioral or performance measures was not examined in the present study.

Critically, random node failures produced substantially smaller differences across emotional states compared to targeted attacks, revealing an important asymmetry in network architecture across emotional conditions. This differential vulnerability pattern suggests that network organization during different emotional states differs strategically around critical hub nodes rather than being uniformly distributed throughout the network. Targeted attacks, which sequentially remove nodes with highest betweenness centrality, specifically probe the network's dependence on critical connector hubs that bridge distinct functional modules and facilitate inter-regional communication (Albert et al., 2000; Lo et al., 2015). The pronounced resilience differences observed across emotional states under targeted attacks indicate that networks during different emotional conditions differ in the redundancy, connectivity patterns, and hierarchical organization surrounding these strategically important hub regions, rather than simply differing in global connection density or peripheral network properties (Lo et al., 2015; Bullmore and Sporns, 2009). This hub-centric pattern of differences suggests that networks during different emotional states show distinct configurations of the most critical communication pathways and bottleneck regions while exhibiting relatively similar less central, peripheral connections relatively unchanged, thereby potentially maximizing functional efficiency while minimizing metabolic costs of widespread network reconfiguration, though the functional consequences of these topological differences remain to be validated through concurrent behavioral and physiological measurements.

These findings reveal differences in brain network resilience across emotional conditions, demonstrating that emotional context is associated with changes in not only transient patterns of regional neural activity but also the fundamental structural robustness and topological organization of large-scale functional connectivity patterns. This dynamic network reorganization may represent a core mechanism underlying successful emotion regulation, where the brain strategically redistributes connectivity to maintain adaptive functioning under varying affective demands, though this functional interpretation requires validation through concurrent emotion regulation tasks and behavioral outcomes. The observed frequency-specific resilience profiles suggest potential implications for understanding both healthy emotion regulation mechanisms and the pathophysiology of affective disorders, though these implications should be interpreted cautiously as testable hypotheses rather than established conclusions. Our study examined only neurotypical young adults experiencing brief VR-induced emotions without clinical populations, longitudinal assessments, or direct validation against mental health outcomes. Disrupted network resilience, particularly altered hub connectivity or loss of frequency-specific optimization patterns during emotional challenges, may be associated with dysfunctional emotional processing (Lo et al., 2015; Pessoa, 2008), though this hypothesis requires validation in clinical samples. Future studies integrating behavioral measures, investigating individual differences in emotional reactivity, or employing cross-frequency coupling analyses could further elucidate the mechanisms underlying the relationship between emotional states and adaptive or maladaptive network resilience. Critical next steps include examining whether network resilience patterns differ in individuals with affective disorders, whether these metrics predict clinical vulnerability or treatment response longitudinally, and whether interventions targeting emotion regulation modify resilience profiles. Only such direct clinical validation can establish whether network resilience represents a clinically actionable biomarker rather than an interesting but clinically unvalidated neural correlate of normative emotional processing. Additionally, examining how these resilience patterns evolve during sustained emotional experiences or emotion regulation strategies could provide insights into the dynamic plasticity of emotional brain networks.

### 4.2 Limitations, methodological considerations, and future work

We acknowledge several limitations that could be addressed in future studies. A methodological limitation of this study concerns the brief duration of VR stimuli and analysis epochs (4 s). While these dynamic, immersive VR stimuli enhance ecological validity compared to traditional 2D presentations and can elicit rapid emotional responses, the short epoch length constrains the reliability of coherence estimates in lower frequency bands. Specifically, delta (1–4 Hz) oscillations complete only 4–16 cycles within a 4-s window, approaching the minimum requirements for stable coherence estimation (Nunez et al., 1997). This limited temporal resolution may reduce estimate stability and increase sensitivity to non-stationarities in delta and theta bands compared to higher frequencies where more oscillatory cycles are captured. We partially addressed this through cross-trial averaging within emotional conditions, which improves estimate reliability (Bastos and Schoffelen, 2015). However, we interpret low-frequency findings conservatively, whereas our main conclusions emphasize the more robust and well-powered effects observed in alpha and higher frequency bands. Future studies would benefit from longer stimulus presentations, continuous naturalistic VR scenarios, or alternative connectivity measures [e.g., phase lag index (Stam et al., 2007), weighted phase lag index (Vinck et al., 2011), imaginary coherence (Nolte et al., 2004)] that accommodate non-stationarity and shorter time windows.

Our sensor-space analysis has inherent limitations related to volume conduction, where activity from a single neural source propagates to multiple scalp electrodes, potentially creating spurious connectivity estimates (Schoffelen and Gross, 2009). Coherence-based connectivity is particularly susceptible to these zero-lag artifacts, which may inflate connectivity strength and affect the spatial specificity of identified hub nodes. Alternative connectivity measures such as weighted phase lag index (wPLI) or imaginary coherence that exclude instantaneous correlations would reduce sensitivity to volume conduction (Vinck et al., 2011; Nolte et al., 2004). However, our comparative approach focusing on relative network topology differences across emotional conditions partially mitigates these concerns, as volume conduction artifacts should affect all conditions similarly under stable recording conditions. Nevertheless, source-space analyses using individualized head models would provide more anatomically precise characterization of emotion-dependent network resilience and should be priorities for future research.

Our use of minimum spanning tree (MST) for network construction has important interpretive implications. While MST provides a principled, threshold-free approach ensuring network connectedness (Stam et al., 2014), it enforces an acyclic tree topology that eliminates redundant pathways and cycles—features fundamental to functional brain network resilience (van Wijk et al., 2010). Consequently, our resilience metrics may partially reflect MST structural constraints rather than purely intrinsic emotional network properties. The observed emotional state differences could arise because emotions modulate edge weights (leading MST to select different pathways) or could reflect MST-imposed topology. Supplementary analyses using proportional thresholding (retaining top 8.67% of edges without MST constraint) showed qualitatively consistent patterns, suggesting our findings are not purely MST artifacts, though this does not fully resolve the concern. Future studies should employ multiple network construction methods (Tumminello et al., 2005; Zalesky et al., 2010; Barrat et al., 2004), including weighted approaches that preserve all connections, to confirm the robustness of emotion-resilience relationships across methodological choices.

Our emotion classification relied on self-reported valence ratings without independent validation of neural separability across emotional categories. While self-report is the gold standard for capturing subjective emotional experience (Mauss and Robinson, 2009), demonstrating that behaviourally-defined categories correspond to distinct neural states through classification analyses (e.g., decoding emotional states from EEG spectral or connectivity features) would strengthen confidence in our findings. The systematic network resilience differences we observed across emotional conditions provide indirect evidence of neural separability. Nevertheless, future studies employing machine learning classification to validate neural distinctiveness of emotional states, potentially using the graph-theoretical metrics themselves as features, would complement our network resilience characterization and establish whether these metrics provide sufficient information for emotion recognition applications (Jo et al., 2025).

The enhanced high-gamma resilience during negative emotions should be interpreted cautiously due to known EEG limitations at high frequencies. High-gamma band signals (50–80 Hz) are susceptible to contamination from facial and neck muscle artifacts (Muthukumaraswamy, 2013), which may differ across emotional states. Additionally, high-frequency neural oscillations attenuate more strongly at the scalp, reducing signal reliability compared to lower frequencies (Whitham et al., 2007). While our preprocessing addressed these issues through artifact rejection and ICA, residual EMG contamination cannot be entirely excluded. Therefore, the observed high-gamma patterns could reflect genuine neural local processing stabilization during aversive processing, differential EMG contamination across emotional states, or some combination. Validation through intracranial recordings, source reconstruction, or combined EEG-EMG approaches would clarify the neural vs. artifactual contributions to a (McMenamin et al., 2010; Subramanian et al., 2025).

Our study did not include behavioral or performance measures beyond self-reported valence ratings, limiting our ability to link network resilience to functional outcomes. We cannot determine whether observed resilience differences predict emotion regulation capacity, cognitive flexibility, or adaptive emotional responses. Future studies should integrate behavioral tasks (e.g., emotion regulation, cognitive performance under emotional contexts) to establish whether network resilience patterns have functionally-relevant consequences and predict individual differences in emotional and cognitive capacities.

## Data Availability

Publicly available datasets were analyzed in this study. This data can be found here: This study is based on secondary analysis of the publicly available VREED dataset originally published by Yu et al. (2022). The VREED dataset is available from Dr. Yingjie Li (liyj@i.shu.edu.cn), the corresponding author of the original publication, upon reasonable request and subject to data usage agreements. All data used in this study were obtained in accordance with the original dataset's terms of use and ethical approval.

## References

[B1] AchardS. BullmoreE. (2007). Efficiency and cost of economical brain functional networks. PLoS Comput. Biol. 3:e17. doi: 10.1371/journal.pcbi.003001717274684 PMC1794324

[B2] Albert R. Jeong H. Barabási A.-L. (2000). Error and attack tolerance of complex networks. Nature 406, 378–82. doi: 10.1038/35019019 10935628

[B3] AljalalM. AldosariS. A. MolinasM. AlSharabiK. AlturkiF. A. (2022). Detection of Parkinson's disease from EEG signals using discrete wavelet transform, different entropy measures, and machine learning techniques. Sci. Rep. 12:22547. doi: 10.1038/s41598-022-26644-736581646 PMC9800369

[B4] Balconi M. Lucchiari C. (2008). Consciousness and arousal effects on emotional face processing as revealed by brain oscillations. A gamma band analysis. Int. J. Psychophysiol. 67, 41–46. doi: 10.1016/j.ijpsycho.2007.10.002 17997495

[B5] Barrat A. Barthélemy M. Vespignani A. (2004). Weighted evolving networks: coupling topology and weight dynamics. Phys. Rev. Lett. 92:228701. doi: 10.1103/PhysRevLett.92.228701 15245264

[B6] Basharpoor S. Heidari F. Molavi P. (2021). EEG coherence in theta, alpha, and beta bands in frontal regions and executive functions. Appl. Neuropsychol. Adult 28, 310–317. doi: 10.1080/23279095.2019.1632860 31282216

[B7] BassettD. S. BullmoreE. VerchinskiB. A. MattayV. S. WeinbergerD. R. Meyer-LindenbergA. (2008). Hierarchical organization of human cortical networks in health and schizophrenia. J. Neurosci. 28, 9239–9248. doi: 10.1523/JNEUROSCI.1929-08.200818784304 PMC2878961

[B8] BastosA. M. SchoffelenJ. M. (2015). A tutorial review of functional connectivity analysis methods and their interpretational pitfalls. Front. Syst. Neurosci. 9:175. doi: 10.3389/fnsys.2015.0017526778976 PMC4705224

[B9] BaumgartnerT. SpeckD. WettsteinD. MasnariO. BeeliG. JänckeL. (2008). Feeling present in arousing virtual reality worlds: prefrontal brain regions differentially orchestrate presence experience in adults and children. Front. Hum. Neurosci. 2:8. doi: 10.3389/neuro.09.008.200818958209 PMC2572200

[B10] Benjamini Y. Hochberg Y. (1995). Controlling the false discovery rate: a practical and powerful approach to multiple testing. J. R. Stat. Soc. Ser. B Methodol. 57, 289–300. doi: 10.1111/j.2517-6161.1995.tb02031.x

[B11] Bevilacqua D. Davidesco I. Wan L. Chaloner K. Rowland J. Ding M. . (2019). Brain-to-brain synchrony and learning outcomes vary by student–teacher dynamics: evidence from a real-world classroom electroencephalography study. J. Cogn. Neurosci. 31, 401–411. doi: 10.1162/jocn_a_01274 29708820

[B12] Bowyer S. M. (2016). Coherence a measure of the brain networks: past and present. Neuropsychiatr. Electrophysiol. 2:1. doi: 10.1186/s40810-015-0015-7

[B13] Bradley M. M. Lang P. J. (2007). “Emotion and motivation,” in Handbook of Psychophysiology, 3rd Edn., eds. J. T. Cacioppo, L. G. Tassinary, and G. G. Berntson (Cambridge University Press), 581–607. doi: 10.1017/CBO9780511546396.025

[B14] Bullmore E. Sporns O. (2009). Complex brain networks: graph theoretical analysis of structural and functional systems. Nat. Rev. Neurosci. 10, 186–98. doi: 10.1038/nrn2575 19190637

[B15] BuzsákiG. WangX.-J. (2012). Mechanisms of gamma oscillations. Annu. Rev. Neurosci. 35, 203–25. doi: 10.1146/annurev-neuro-062111-15044422443509 PMC4049541

[B16] Cabral J. Kringelbach M. Deco G. (2012). Functional graph alterations in schizophrenia: a result from a global anatomic decoupling? Pharmacopsychiatry 45, S57–S64. doi: 10.1055/s-0032-1309001 22565236

[B17] CaoR. HaoY. WangX. GaoY. ShiH. HuoS. . (2020). EEG functional connectivity underlying emotional valance and arousal using minimum spanning trees. Front. Neurosci. 14:355. doi: 10.3389/fnins.2020.0035532457566 PMC7222391

[B18] Cisler J. James G. Tripathi S. Mletzko T. Heim C. Hu X. . (2013). Differential functional connectivity within an emotion regulation neural network among individuals resilient and susceptible to the depressogenic effects of early life stress. Psychol. Med. 43, 507–518. doi: 10.1017/S0033291712001390 22781311

[B19] Cohen J. (1977). Statistical Power Analysis for the Behavioral Sciences. Hillsdale, NJ: Lawrence Erlbaum Associates, Inc.

[B20] CohenJ. R. D'EspositoM. (2016). The segregation and integration of distinct brain networks and their relationship to cognition. J. Neurosci. 36, 12083–12094. doi: 10.1523/JNEUROSCI.2965-15.201627903719 PMC5148214

[B21] Crone N. E. Sinai A. Korzeniewska A. (2006). High-frequency gamma oscillations and human brain mapping with electrocorticography. Prog. Brain Res. 159, 275–295. doi: 10.1016/S0079-6123(06)59019-3 17071238

[B22] Delorme A. Makeig S. (2004). EEGLAB: an open source toolbox for analysis of single-trial EEG dynamics including independent component analysis. J. Neurosci. Methods 134, 9–21. doi: 10.1016/j.jneumeth.2003.10.009 15102499

[B23] FischerA. S. CamachoM. C. HoT. C. Whitfield-GabrieliS. GotlibI. H. (2018). Neural markers of resilience in adolescent females at familial risk for major depressive disorder. JAMA Psychiatry 75, 493–502. doi: 10.1001/jamapsychiatry.2017.451629562053 PMC5875355

[B24] Fornito A. Zalesky A. Bullmore E. (2016). Fundamentals of Brain Network Analysis. San Diego, CA: Academic Press.

[B25] FredricksonB. L. (2001). The role of positive emotions in positive psychology: the broaden-and-build theory of positive emotions. Am. Psychol. 56:218. doi: 10.1037//0003-066X.56.3.21811315248 PMC3122271

[B26] FredricksonB. L. BraniganC. (2005). Positive emotions broaden the scope of attention and thought-action repertoires. Cogn. Emot. 19, 313–332. doi: 10.1080/0269993044100023821852891 PMC3156609

[B27] Fries P. (2009). Neuronal gamma-band synchronization as a fundamental process in cortical computation. Annu. Rev. Neurosci. 32, 209–224. doi: 10.1146/annurev.neuro.051508.135603 19400723

[B28] Games P. A. Howell J. F. (1976). Pairwise multiple comparison procedures with unequal n's and/or variances: a Monte Carlo study. J. Educ. Stat. 1, 113–125. doi: 10.3102/10769986001002113

[B29] Gonzalez-EscamillaG. MuthuramanM. ChirumamillaV. C. VogtJ. GroppaS. (2018). Brain networks reorganization during maturation and healthy aging-emphases for resilience. Front. Psychiatry 9:601. doi: 10.3389/fpsyt.2018.0060130519196 PMC6258799

[B30] HeY. ChenZ. EvansA. (2008). Structural insights into aberrant topological patterns of large-scale cortical networks in Alzheimer's disease. J. Neurosci. 28, 4756–4766. doi: 10.1523/JNEUROSCI.0141-08.200818448652 PMC6670444

[B31] HofmannS. M. KlotzscheF. MariolaA. NikulinV. VillringerA. GaeblerM. (2021). Decoding subjective emotional arousal from EEG during an immersive virtual reality experience. eLife 10:e64812. doi: 10.7554/eLife.6481234708689 PMC8673835

[B32] Isen A. M. Rosenzweig A. S. Young M. J. (1991). The influence of positive affect on clinical problem solving. Med. Decis. Making 11, 221–227. doi: 10.1177/0272989X9101100313 1881279

[B33] Ismail L. E. Karwowski W. (2020). A graph theory-based modeling of functional brain connectivity based on EEG: a systematic review in the context of neuroergonomics. IEEE Access 8, 155103–155135. doi: 10.1109/ACCESS.2020.3018995

[B34] JoH. J. LeeM. J. LeeW. H. (2025). Graph-theoretical analysis of EEG-based functional connectivity during emotional experience in virtual reality for emotion recognition. Sci. Rep. 15:39965. doi: 10.1038/s41598-025-23573-z41238618 PMC12618519

[B35] JoyceK. E. HayasakaS. LaurientiP. J. (2013). The human functional brain network demonstrates structural and dynamical resilience to targeted attack. PLoS Comput. Biol. 9:e1002885. doi: 10.1371/journal.pcbi.100288523358557 PMC3554573

[B36] Jurcak V. Tsuzuki D. Dan I. (2007). 10/20, 10/10, and 10/5 systems revisited: their validity as relative head-surface-based positioning systems. NeuroImage 34, 1600–1611. doi: 10.1016/j.neuroimage.2006.09.024 17207640

[B37] KassamK. S. MarkeyA. R. CherkasskyV. L. LoewensteinG. JustM. A. (2013). Identifying emotions on the basis of neural activation. PLoS ONE 8:e66032. doi: 10.1371/journal.pone.006603223840392 PMC3686858

[B38] KimT. K. (2017). Understanding one-way ANOVA using conceptual figures. Korean J. Anesthesiol. 70:22. doi: 10.4097/kjae.2017.70.1.2228184262 PMC5296382

[B39] Klimesch W. Sauseng P. Hanslmayr S. (2007). EEG alpha oscillations: the inhibition–timing hypothesis. Brain Res. Rev. 53, 63–88. doi: 10.1016/j.brainresrev.2006.06.003 16887192

[B40] LeeW. H. BullmoreE. FrangouS. (2017). Quantitative evaluation of simulated functional brain networks in graph theoretical analysis. NeuroImage 146, 724–733. doi: 10.1016/j.neuroimage.2016.08.05027568060 PMC5312789

[B41] LeeY.-Y. HsiehS. (2014). Classifying different emotional states by means of EEG-based functional connectivity patterns. PLoS ONE 9:e95415. doi: 10.1371/journal.pone.009541524743695 PMC3990628

[B42] LiuP. ShenH. JiS. (2019). Functional connectivity pattern analysis underlying neural oscillation synchronization during deception. Neural Plast. 2019:2684821. doi: 10.1155/2019/268482130906317 PMC6393932

[B43] LoC.-Y. Z. SuT.-W. HuangC.-C. HungC.-C. ChenW.-L. LanT.-H. . (2015). Randomization and resilience of brain functional networks as systems-level endophenotypes of schizophrenia. Proc. Natl. Acad. Sci. U. S. A. 112, 9123–9128. doi: 10.1073/pnas.150205211226150519 PMC4517199

[B44] LynallM.-E. BassettD. S. KerwinR. McKennaP. J. KitzbichlerM. MullerU. . (2010). Functional connectivity and brain networks in schizophrenia. J. Neurosci. 30, 9477–9487. doi: 10.1523/JNEUROSCI.0333-10.201020631176 PMC2914251

[B45] Marín-MoralesJ. LlinaresC. GuixeresJ. AlcañizM. (2020). Emotion recognition in immersive virtual reality: from statistics to affective computing. Sensors 20:5163. doi: 10.3390/s2018516332927722 PMC7570837

[B46] Martini N. Menicucci D. Sebastiani L. Bedini R. Pingitore A. Vanello N. . (2012). The dynamics of EEG gamma responses to unpleasant visual stimuli: from local activity to functional connectivity. NeuroImage 60, 922–932. doi: 10.1016/j.neuroimage.2012.01.060 22270349

[B47] MaussI. B. RobinsonM. D. (2009). Measures of emotion: a review. Cogn. Emot. 23, 209–237. doi: 10.1080/0269993080220467719809584 PMC2756702

[B48] McMenaminB. W. ShackmanA. J. MaxwellJ. S. BachhuberD. R. W. KoppenhaverA. M. GreischarL. L. . (2010). Validation of ICA-based myogenic artifact correction for scalp and source-localized EEG. NeuroImage 49, 2416–2432. doi: 10.1016/j.neuroimage.2009.10.01019833218 PMC2818255

[B49] Mitchell D. G. (2011). The nexus between decision making and emotion regulation: a review of convergent neurocognitive substrates. Behav. Brain Res. 217, 215–231. doi: 10.1016/j.bbr.2010.10.030 21055420

[B50] MuthukumaraswamyS. D. (2013). High-frequency brain activity and muscle artifacts in MEG/EEG: a review and recommendations. Front. Hum. Neurosci. 7:138. doi: 10.3389/fnhum.2013.0013823596409 PMC3625857

[B51] Nolte G. Bai O. Wheaton L. Mari Z. Vorbach S. Hallett M. (2004). Identifying true brain interaction from EEG data using the imaginary part of coherency. Clin. Neurophysiol. 115, 2292–2307. doi: 10.1016/j.clinph.2004.04.029 15351371

[B52] Nunez P. L. Srinivasan R. Westdorp A. F. Wijesinghe R. S. Tucker D. M. Silberstein R. B. . (1997). EEG coherency: I: statistics, reference electrode, volume conduction, Laplacians, cortical imaging, and interpretation at multiple scales. Electroencephalogr. Clin. Neurophysiol. 103, 499–515. doi: 10.1016/S0013-4694(97)00066-7 9402881

[B53] PabanV. ModoloJ. MheichA. HassanM. (2019). Psychological resilience correlates with EEG source-space brain network flexibility. Netw. Neurosci. 3, 539–50. doi: 10.1162/netn_a_0007930984906 PMC6444909

[B54] Palva S. Palva J. M. (2007). New vistas for α-frequency band oscillations. Trends Neurosci. 30, 150–158. doi: 10.1016/j.tins.2007.02.001 17307258

[B55] PalvaS. PalvaJ. M. (2011). Functional roles of alpha-band phase synchronization in local and large-scale cortical networks. Front. Psychol. 2:204. doi: 10.3389/fpsyg.2011.0020421922012 PMC3166799

[B56] Pessoa L. (2008). On the relationship between emotion and cognition. Nat. Rev. Neurosci. 9, 148–158. doi: 10.1038/nrn2317 18209732

[B57] PhelpsE. A. LingS. CarrascoM. (2006). Emotion facilitates perception and potentiates the perceptual benefits of attention. Psychol. Sci. 17, 292–299. doi: 10.1111/j.1467-9280.2006.01701.x16623685 PMC1555625

[B58] RayS. MaunsellJ. H. (2011a). Different origins of gamma rhythm and high-gamma activity in macaque visual cortex. PLoS Biol. 9:e1000610. doi: 10.1371/journal.pbio.100061021532743 PMC3075230

[B59] RayS. MaunsellJ. H. (2011b). Network rhythms influence the relationship between spike-triggered local field potential and functional connectivity. J. Neurosci. 31, 12674–82. doi: 10.1523/JNEUROSCI.1856-11.201121880928 PMC3488382

[B60] Richter-Levin G. Akirav I. (2003). Emotional tagging of memory formation—in the search for neural mechanisms. Brain Res. Rev. 43, 247–256. doi: 10.1016/j.brainresrev.2003.08.005 14629927

[B61] Rodríguez A. Rey B. Clemente M. Wrzesien M. Alcañiz M. (2015). Assessing brain activations associated with emotional regulation during virtual reality mood induction procedures. Expert Syst. Appl. 42, 1699–1709. doi: 10.1016/j.eswa.2014.10.006

[B62] Rubinov M. Sporns O. (2010). Complex network measures of brain connectivity: uses and interpretations. NeuroImage 52, 1059–1069. doi: 10.1016/j.neuroimage.2009.10.003 19819337

[B63] Russell J. A. (1980). A circumplex model of affect. J. Pers. Soc. Psychol. 39, 1161–1178. doi: 10.1037/h0077714

[B64] Sadaghiani S. Kleinschmidt A. (2016). Brain networks and α-oscillations: structural and functional foundations of cognitive control. Trends Cogn. Sci. 20, 805–817. doi: 10.1016/j.tics.2016.09.004 27707588

[B65] SchoffelenJ. M. GrossJ. (2009). Source connectivity analysis with MEG and EEG. Hum. Brain Mapp. 30, 1857–1865. doi: 10.1002/hbm.2074519235884 PMC6870611

[B66] SpornsO. (2018). Graph theory methods: applications in brain networks. Dialogues Clin. Neurosci. 20, 111–121. doi: 10.31887/DCNS.2018.20.2/osporns30250388 PMC6136126

[B67] StamC. J. NolteG. DaffertshoferA. (2007). Phase lag index: assessment of functional connectivity from multi channel EEG and MEG with diminished bias from common sources. Hum. Brain Mapp. 28, 1178–1193. doi: 10.1002/hbm.2034617266107 PMC6871367

[B68] Stam C. J. Tewarie P. Van Dellen E. van Straaten E. C. Hillebrand A. Van Mieghem P. (2014). The trees and the forest: characterization of complex brain networks with minimum spanning trees. Int. J. Psychophysiol. 92, 129–138. doi: 10.1016/j.ijpsycho.2014.04.001 24726900

[B69] SubramanianA. K. TalbotA. KimN. ParmigianiS. ClineC. C. SolomonE. A. . (2025). Scalp EEG predicts intracranial brain activity in humans. bioRxiv [Preprint]. 2025.04.07.647612. doi: 10.1101/2025.04.07.64761240291696 PMC12026988

[B70] TumminelloM. AsteT. Di MatteoT. MantegnaR. N. (2005). A tool for filtering information in complex systems. Proc. Natl. Acad. Sci. U. S. A. 102, 10421–10426. doi: 10.1073/pnas.050029810216027373 PMC1180754

[B71] van WijkB. C. M. StamC. J. DaffertshoferA. (2010). Comparing brain networks of different size and connectivity density using graph theory. PLoS ONE 5:e13701. doi: 10.1371/journal.pone.001370121060892 PMC2965659

[B72] Vinck M. Oostenveld R. van Wingerden M. Battaglia F. Pennartz C. M. A. (2011). An improved index of phase-synchronization for electrophysiological data in the presence of volume-conduction, noise and sample-size bias. NeuroImage 55, 1548–1565. doi: 10.1016/j.neuroimage.2011.01.055 21276857

[B73] Wang Z.-M. Zhou R. He Y. Guo X.-M. (2020). Functional integration and separation of brain network based on phase locking value during emotion processing. IEEE Trans. Cogn. Dev. Syst. 15, 444–453. doi: 10.1109/TCDS.2020.3001642

[B74] Whitham E. M. Pope K. J. Fitzgibbon S. P. Lewis T. Clark C. R. Loveless S. . (2007). Scalp electrical recording during paralysis: quantitative evidence that EEG frequencies above 20 Hz are contaminated by EMG. Clin. Neurophysiol. 118, 1877–1888. doi: 10.1016/j.clinph.2007.04.027 17574912

[B75] Yu M. Xiao S. Hua M. Wang H. Chen X. Tian F. . (2022). EEG-based emotion recognition in an immersive virtual reality environment: from local activity to brain network features. Biomed. Signal Process. Control 72:103349. doi: 10.1016/j.bspc.2021.103349

[B76] Zalesky A. Fornito A. Bullmore E. T. (2010). Network-based statistic: identifying differences in brain networks. NeuroImage 53, 1197–1207. doi: 10.1016/j.neuroimage.2010.06.041 20600983

