# Supplementary material for: Topological resilience of EEG-based functional networks during virtual reality-induced emotional states
Source: Front Hum Neurosci. 2026 Apr 10;20:1777965. doi: 10.3389/fnhum.2026.1777965 (PMC13106194; doi:10.3389/fnhum.2026.1777965)
Supplement: Supplementary file 1 [file Data_Sheet_1.docx]

Supplementary Material


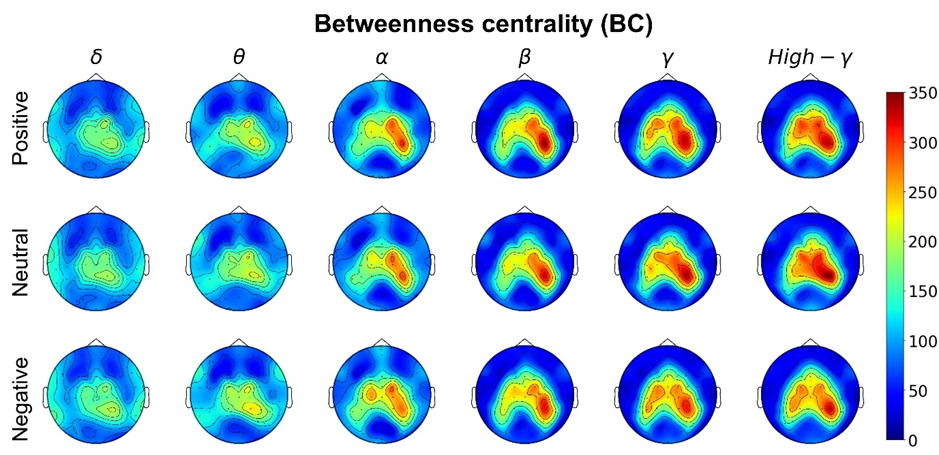


**Supplementary Figure 1.** Topographical maps showing betweenness centrality (BC) for each emotional state (positive, neutral, and negative) across different frequency bands. Each panel displays the average value for betweenness centrality across all participants in a particular frequency band (e.g., delta). $\delta$: delta; $\theta$: theta; $\alpha$: alpha; $\beta$: beta; $\gamma$: gamma; $High-\gamma$: high gamma.


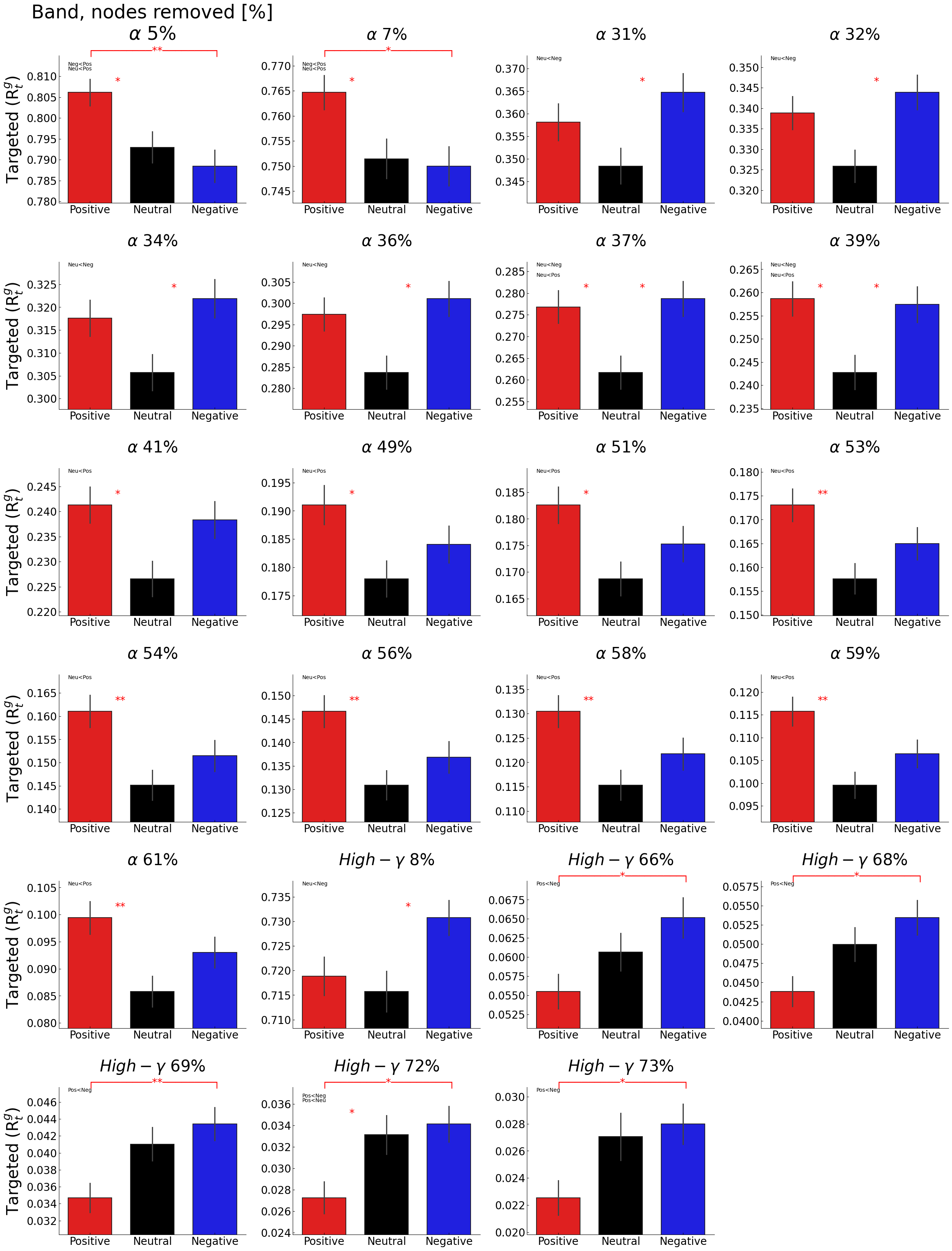


**Supplementary Figure S2.** The mean and standard error of mean (SEM) of network resilience to targeted attacks (global efficiency, $R_{t}^{g}$) across all participants for different frequency bands at specific connection densities (%). Network resilience values that show statistically significant differences between different emotional groups are only shown at selected connection densities (FDR-corrected p-value < 0.05). Red asterisks indicate statistically significant differences between emotional groups.
(*: FDR-corrected p-value < 0.05, **: FDR-corrected p-value < 0.01, ***: FDR-corrected p-value < 0.001). $\delta$: delta; $\theta$: theta; $\alpha$: alpha; $\beta$: beta; $\gamma$: gamma; $High-\gamma$: high gamma.

***
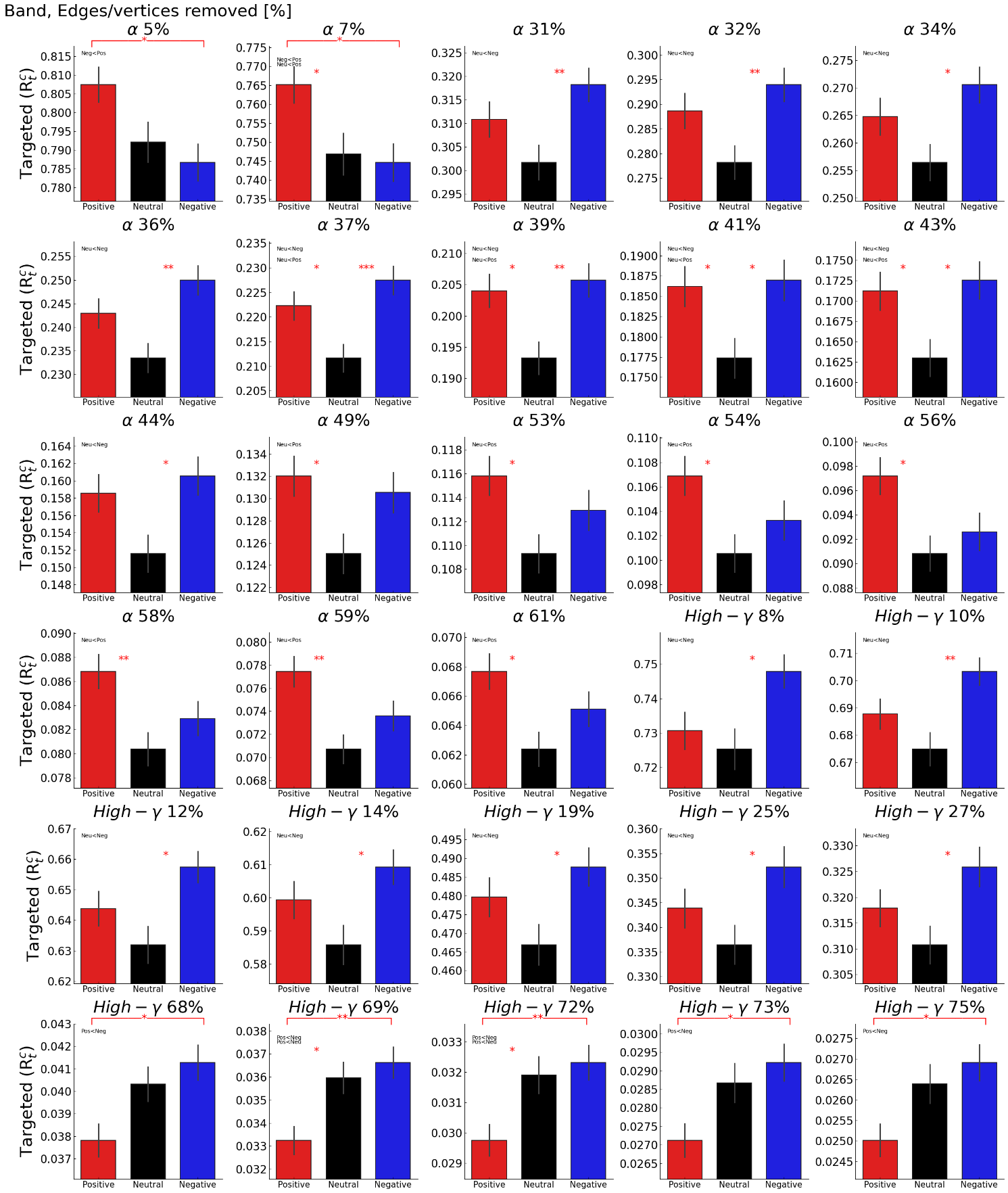
***

**Supplementary Figure S3.** The mean and standard error of mean (SEM) of network resilience to targeted attacks (largest connected component, $R_{t}^{c}$) across all participants for different frequency bands at specific connection densities (%). Network resilience values that show statistically significant differences between different emotional groups are only shown at selected connection densities (FDR-corrected p-value < 0.05). Red asterisks indicate statistically significant differences between emotional groups. (*: FDR-corrected p-value < 0.05, **: FDR-corrected p-value < 0.01, ***: FDR-corrected p-value < 0.001). $\delta$: delta; $\theta$: theta; $\alpha$: alpha; $\beta$: beta; $\gamma$: gamma; $High-\gamma$: high gamma.


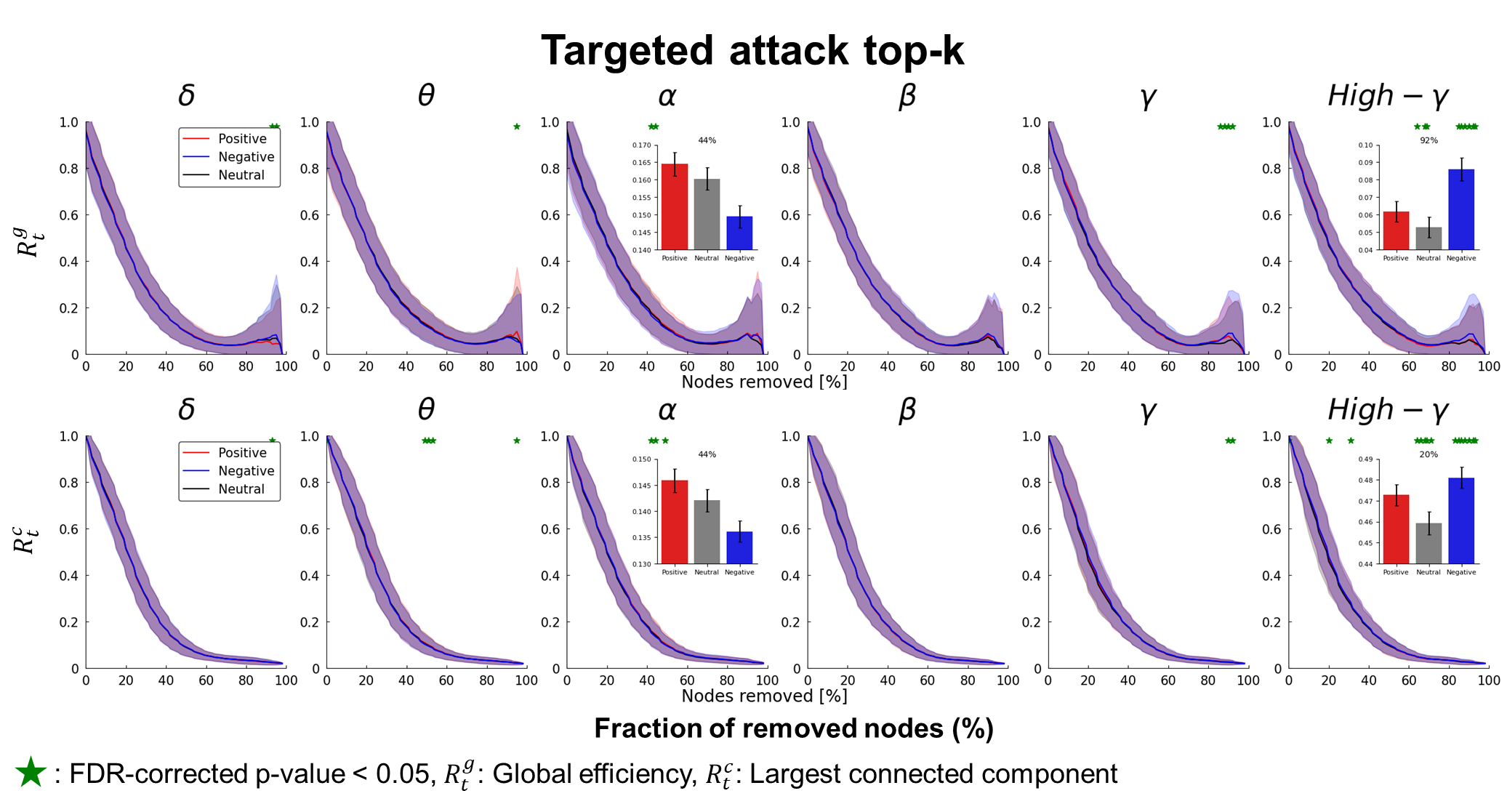


**Supplementary Figure S4.** Network resilience under targeted attacks (top-k proportional thresholding) varies across emotional states and frequency bands. Resilience profiles comparing positive (red), negative (blue), and neutral (black) emotional states during progressive targeted removal of high-betweenness centrality nodes across six frequency bands (δ: delta, θ: theta, α: alpha, β: beta, γ: gamma, High-γ: high gamma). (a) Global efficiency ($R_{t}^{g}$) quantifies network integration and information transfer efficiency as a function of nodes removed. (b) Largest connected component size ($R_{t}^{c}$) indicates network fragmentation resistance. Green dots mark fractions of removed nodes where emotional states show statistically significant differences (FDR-corrected p < 0.05). Inset panels display detailed comparisons at specific removal fractions (%) where the most pronounced significant differences between emotional conditions are observed. Error bands represent the standard deviation across participants.


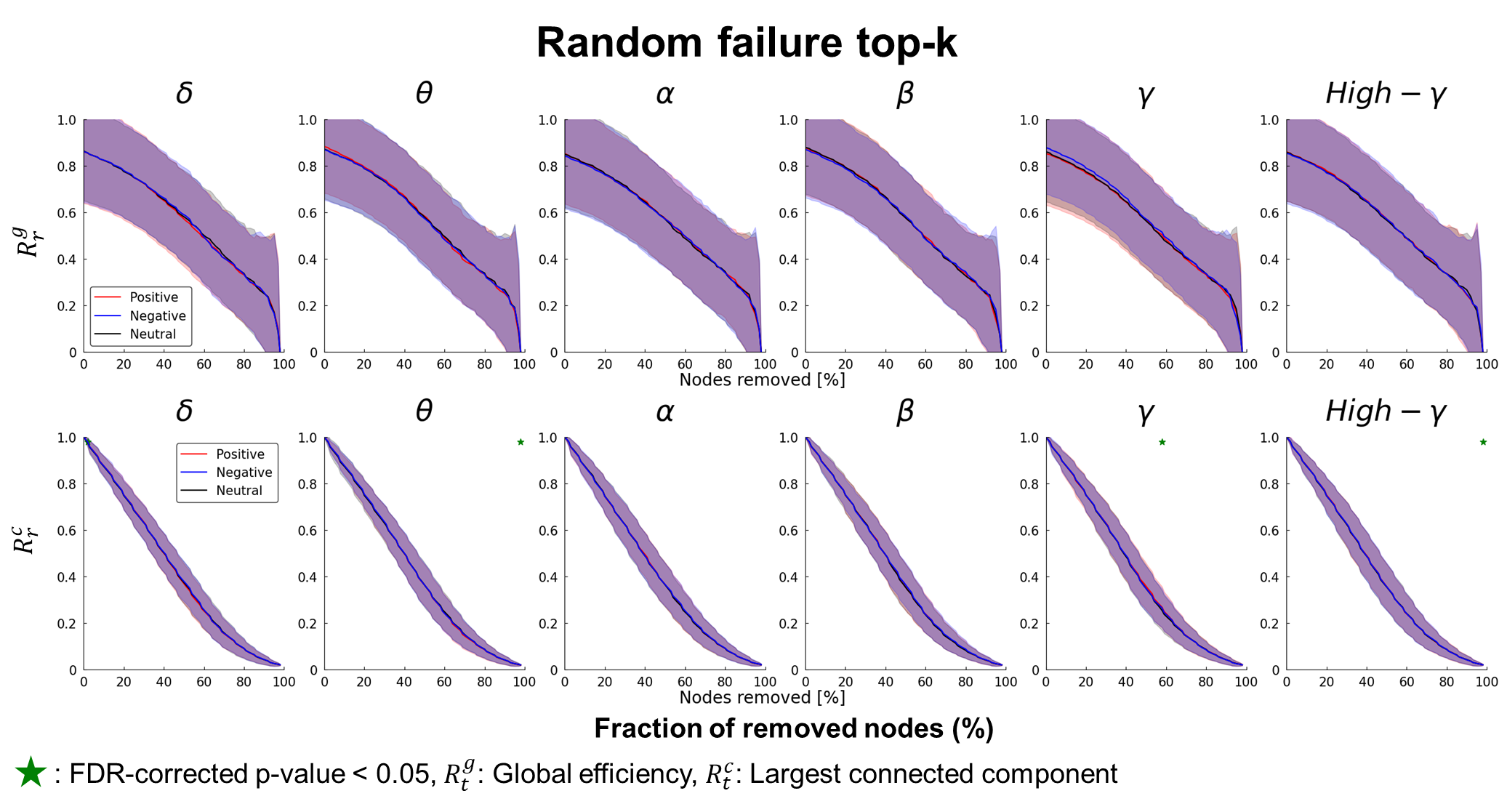


**Supplementary Figure S5.** Network resilience under random failure (top-k proportional thresholding) varies across emotional states and frequency bands. Resilience profiles comparing positive (red), negative (blue), and neutral (black) emotional states during progressive targeted removal of high-betweenness centrality nodes across six frequency bands (δ: delta, θ: theta, α: alpha, β: beta, γ: gamma, High-γ: high gamma). (a) Global efficiency ($R_{t}^{g}$) quantifies network integration and information transfer efficiency as a function of nodes removed. (b) Largest connected component size ($R_{t}^{c}$) indicates network fragmentation resistance. Green dots mark fractions of removed nodes where emotional states show statistically significant differences (FDR-corrected p < 0.05). Inset panels display detailed comparisons at specific removal fractions (%) where the most pronounced significant differences between emotional conditions are observed. Error bands represent the standard deviation across participants.

**Supplementary Table S1**. Effect sizes for resilience-curve differences in global efficiency (GE) under targeted attacks. Paired Cohen’s d and 95% confidence intervals are reported for subject-level AUC summaries of GE resilience curves across emotional-condition contrasts (Positive vs Neutral, Positive vs Negative, Neutral vs Negative) for each frequency band (delta–high gamma).

| **Band** | **Comparison** | **Cohen’s_d** | **CI_Lower** | **CI_Upper** |
| --- | --- | --- | --- | --- |
| Delta | Positive_vs_Neutral | 0.188 | -0.298 | 0.674 |
|  | Positive_vs_Negative | 0.343 | -0.153 | 0.839 |
|  | Neutral_vs_Negative | 0.019 | -0.463 | 0.501 |
| Theta | Positive_vs_Neutral | 0.102 | -0.381 | 0.586 |
|  | Positive_vs_Negative | 0.243 | -0.246 | 0.732 |
|  | Neutral_vs_Negative | 0.154 | -0.331 | 0.639 |
| Alpha | Positive_vs_Neutral | 0.491 | -0.019 | 1.001 |
|  | Positive_vs_Negative | 0.065 | -0.418 | 0.547 |
|  | Neutral_vs_Negative | -0.277 | -0.768 | 0.214 |
| Beta | Positive_vs_Neutral | -0.204 | -0.691 | 0.283 |
|  | Positive_vs_Negative | -0.106 | -0.590 | 0.377 |
|  | Neutral_vs_Negative | 0.092 | -0.391 | 0.575 |
| Gamma | Positive_vs_Neutral | -0.074 | -0.556 | 0.409 |
|  | Positive_vs_Negative | -0.141 | -0.626 | 0.343 |
|  | Neutral_vs_Negative | -0.091 | -0.574 | 0.392 |
| High-gamma | Positive_vs_Neutral | 0.047 | -0.435 | 0.529 |
|  | Positive_vs_Negative | -0.256 | -0.746 | 0.234 |
|  | Neutral_vs_Negative | -0.386 | -0.886 | 0.113 |

**Supplementary Table** **S2**. Effect sizes for resilience-curve differences in global efficiency (GE) under random failures. Paired Cohen’s d and 95% confidence intervals are reported for subject-level AUC summaries of GE resilience curves across emotional-condition contrasts (Positive vs Neutral, Positive vs Negative, Neutral vs Negative) for each frequency band (delta–high gamma).

| **Band** | **Comparison** | **Cohen’s_d** | **CI_Lower** | **CI_Upper** |
| --- | --- | --- | --- | --- |
| Delta | Positive_vs_Neutral | 0.034 | -0.448 | 0.516 |
|  | Positive_vs_Negative | 0.164 | -0.321 | 0.649 |
|  | Neutral_vs_Negative | 0.129 | -0.355 | 0.613 |
| Theta | Positive_vs_Neutral | -0.003 | -0.485 | 0.479 |
|  | Positive_vs_Negative | 0.115 | -0.369 | 0.599 |
|  | Neutral_vs_Negative | 0.071 | -0.412 | 0.553 |
| Alpha | Positive_vs_Neutral | 0.027 | -0.455 | 0.509 |
|  | Positive_vs_Negative | -0.099 | -0.582 | 0.384 |
|  | Neutral_vs_Negative | -0.148 | -0.633 | 0.336 |
| Beta | Positive_vs_Neutral | -0.112 | -0.596 | 0.371 |
|  | Positive_vs_Negative | 0.034 | -0.448 | 0.516 |
|  | Neutral_vs_Negative | 0.206 | -0.281 | 0.693 |
| Gamma | Positive_vs_Neutral | 0.101 | -0.382 | 0.584 |
|  | Positive_vs_Negative | 0.089 | -0.394 | 0.572 |
|  | Neutral_vs_Negative | -0.011 | -0.493 | 0.471 |
| High-gamma | Positive_vs_Neutral | -0.001 | -0.483 | 0.481 |
|  | Positive_vs_Negative | -0.156 | -0.641 | 0.329 |
|  | Neutral_vs_Negative | -0.152 | -0.637 | 0.332 |

**Supplementary Table S3**. Effect sizes for resilience-curve differences in largest connected component (LCC) under target attacks. Paired Cohen’s d and 95% confidence intervals are reported for subject-level AUC summaries of LCC resilience curves across emotional-condition contrasts (Positive vs Neutral, Positive vs Negative, Neutral vs Negative) for each frequency band (delta–high gamma).

| **Band** | **Comparison** | **Cohen’s d** | **CI_Lower** | **CI_Upper** |
| --- | --- | --- | --- | --- |
| Delta | Positive_vs_Neutral | 0.193 | -0.293 | 0.679 |
|  | Positive_vs_Negative | 0.347 | -0.150 | 0.843 |
|  | Neutral_vs_Negative | 0.000 | -0.482 | 0.482 |
| Theta | Positive_vs_Neutral | 0.166 | -0.319 | 0.651 |
|  | Positive_vs_Negative | 0.115 | -0.369 | 0.599 |
|  | Neutral_vs_Negative | 0.008 | -0.474 | 0.490 |
| Alpha | Positive_vs_Neutral | 0.323 | -0.171 | 0.818 |
|  | Positive_vs_Negative | 0.094 | -0.389 | 0.578 |
|  | Neutral_vs_Negative | -0.232 | -0.720 | 0.257 |
| Beta | Positive_vs_Neutral | -0.143 | -0.627 | 0.342 |
|  | Positive_vs_Negative | -0.079 | -0.562 | 0.403 |
|  | Neutral_vs_Negative | 0.089 | -0.394 | 0.572 |
| Gamma | Positive_vs_Neutral | 0.031 | -0.451 | 0.513 |
|  | Positive_vs_Negative | -0.059 | -0.542 | 0.423 |
|  | Neutral_vs_Negative | -0.126 | -0.610 | 0.358 |
| High-gamma | Positive_vs_Neutral | 0.184 | -0.302 | 0.671 |
|  | Positive_vs_Negative | -0.243 | -0.732 | 0.246 |
|  | Neutral_vs_Negative | -0.636 | -1.164 | -0.107 |

| **Band** | **Comparison** | **Cohen’s d** | **CI_Lower** | **CI_Upper** |
| --- | --- | --- | --- | --- |
| Delta | Positive_vs_Neutral | -0.068 | -0.550 | 0.415 |
|  | Positive_vs_Negative | 0.022 | -0.460 | 0.504 |
|  | Neutral_vs_Negative | 0.116 | -0.367 | 0.600 |
| Theta | Positive_vs_Neutral | 0.083 | -0.400 | 0.566 |
|  | Positive_vs_Negative | 0.165 | -0.320 | 0.650 |
|  | Neutral_vs_Negative | 0.134 | -0.350 | 0.618 |
| Alpha | Positive_vs_Neutral | -0.034 | -0.516 | 0.449 |
|  | Positive_vs_Negative | 0.071 | -0.411 | 0.554 |
|  | Neutral_vs_Negative | 0.121 | -0.363 | 0.604 |
| Beta | Positive_vs_Neutral | -0.117 | -0.601 | 0.366 |
|  | Positive_vs_Negative | -0.099 | -0.582 | 0.384 |
|  | Neutral_vs_Negative | 0.032 | -0.450 | 0.514 |
| Gamma | Positive_vs_Neutral | -0.020 | -0.502 | 0.462 |
|  | Positive_vs_Negative | -0.059 | -0.542 | 0.423 |
|  | Neutral_vs_Negative | -0.061 | -0.544 | 0.421 |
| High-gamma | Positive_vs_Neutral | 0.175 | -0.311 | 0.660 |
|  | Positive_vs_Negative | -0.168 | -0.653 | 0.317 |
|  | Neutral_vs_Negative | -0.430 | -0.934 | 0.074 |

**Supplementary Table** S4. Effect sizes for resilience-curve differences in largest connected component (LCC) under random failures. Paired Cohen’s d and 95% confidence intervals are reported for subject-level AUC summaries of LCC resilience curves across emotional-condition contrasts (Positive vs Neutral, Positive vs Negative, Neutral vs Negative) for each frequency band (delta–high gamma).
